# Constrictive Pericarditis Presenting as Bilateral Pleural Effusion: A Report of Two Cases

**DOI:** 10.7759/cureus.2451

**Published:** 2018-04-09

**Authors:** Manesh Kumar Gangwani, Salman B Mahmood, Fariha Hasan, Javaid A Khan

**Affiliations:** 1 Medical College, The Aga Khan University, Karachi, PAK; 2 Department of Medicine, Hennepin County Medical Center; 3 Department of Internal Medicine, Dow University of Health Sciences (DUHS), Karachi, Pakistan; 4 Pulmonology, The Aga Khan University, Karachi, PAK

**Keywords:** constrcitive pericarditis, tuberculosis, pleural effusion

## Abstract

Constrictive pericarditis is a rare presentation. We need a very high index of clinical suspicion to diagnose the disease. It most commonly presents secondary to tuberculosis (TB) in the developing world and post-radiation therapy in the developed world. Classically, it presents with symptoms of heart failure and as pericardial thickening or calcification on imaging studies. In hospital settings, constrictive pericarditis is not usually considered as a differential in patients presenting with pleural effusion. According to the literature, associated pleural effusions in cases of constrictive pericarditis could be left-sided. Herein, we present two unusual presentations of cases with bilateral pleural effusions. One of our cases developed constrictive pericarditis with concurrent active tuberculosis. This is a rare presentation because, normally, constrictive pericarditis is a late complication of tuberculosis. We suggest that when dealing with cases of bilateral pleural effusion, the etiology of constrictive pericarditis should be considered.

## Introduction

Constrictive pericarditis is a rare disease that can be a challenge to diagnose unless a high index of suspicion is maintained. Classically, it presents with symptoms of heart failure. On imaging studies, pericardial thickening or calcification may be seen. However, initial diagnostic studies remain non-specific in a significant proportion of cases, for example, chest radiographs show calcifications in only half the cases. In cases of pericardial thickening, an echocardiogram only has 37% sensitivity [1]. Furthermore, the pericardial thickness may be normal in about 20% of patients.

The etiology of this disease has evolved over the years, especially in the Western world, due to an increase in the number of cases secondary to cardiothoracic surgery or previous radiotherapy [2]. In the United States, the incidence of such cases is 800,000 annually. However, in the developing world, tuberculosis (TB) remains the leading cause [2].

Pleural effusion is known to be associated with constrictive pericarditis in up to 55% of cases [3] and is usually left-sided at presentation [4]. Right-sided or bilateral pleural effusions are more characteristic of heart failure. Here, we present two cases of recurrent, unexplained bilateral pleural effusion, which was eventually diagnosed as constrictive pericarditis. Our case also adds to the existing literature by discussing a rare presentation of concurrent active tuberculosis in a patient with constrictive pericarditis.

## Case presentation

Case 1

A 61-year-old male, known asthmatic, presented to the pulmonology clinic with complaints of weight loss for two months and dry cough and dyspnea for three months. His history was positive for TB contact. On examination, he had a pulse of 88 bpm, respiratory rate 22/minute, blood pressure (BP) 130/71, and on auscultation, he had decreased breath sounds on the right side. Radiological imaging revealed a large pleural effusion on the right side and a narrowing of the right main bronchus.

An ultrasound-guided diagnostic thoracentesis was performed and his pleural fluid analysis revealed a protein content of 4.5 g/dl, glucose of 121 mg/dl, lactate dehydrogenase (LDH) of 158 IU/L, and a white blood cell count (WBC) of 200 cells/mm^3^ with 80% lymphocytes. The differential diagnosis included TB and malignancy. Therapeutic thoracentesis was performed and 1450 ml of fluid was drained. The acid-fast bacillus (AFB) smear was positive. Quadruple anti-tuberculosis treatment (ATT) regimen was started for six months. The patient underwent pleural taps four times at another center again.

One week later, the patient presented to the emergency department with hydropneumothorax. Chest tube insertion with lung re-expansion was performed along with the drainage of 1800 ml of fluid. Another 690 ml was drained on a subsequent day. The patient was later discharged and the chest tube was removed in the clinic after resolution of the pneumothorax. The blood sputum culture returned positive for Mycobacterium tuberculosis.

**Figure 1:**
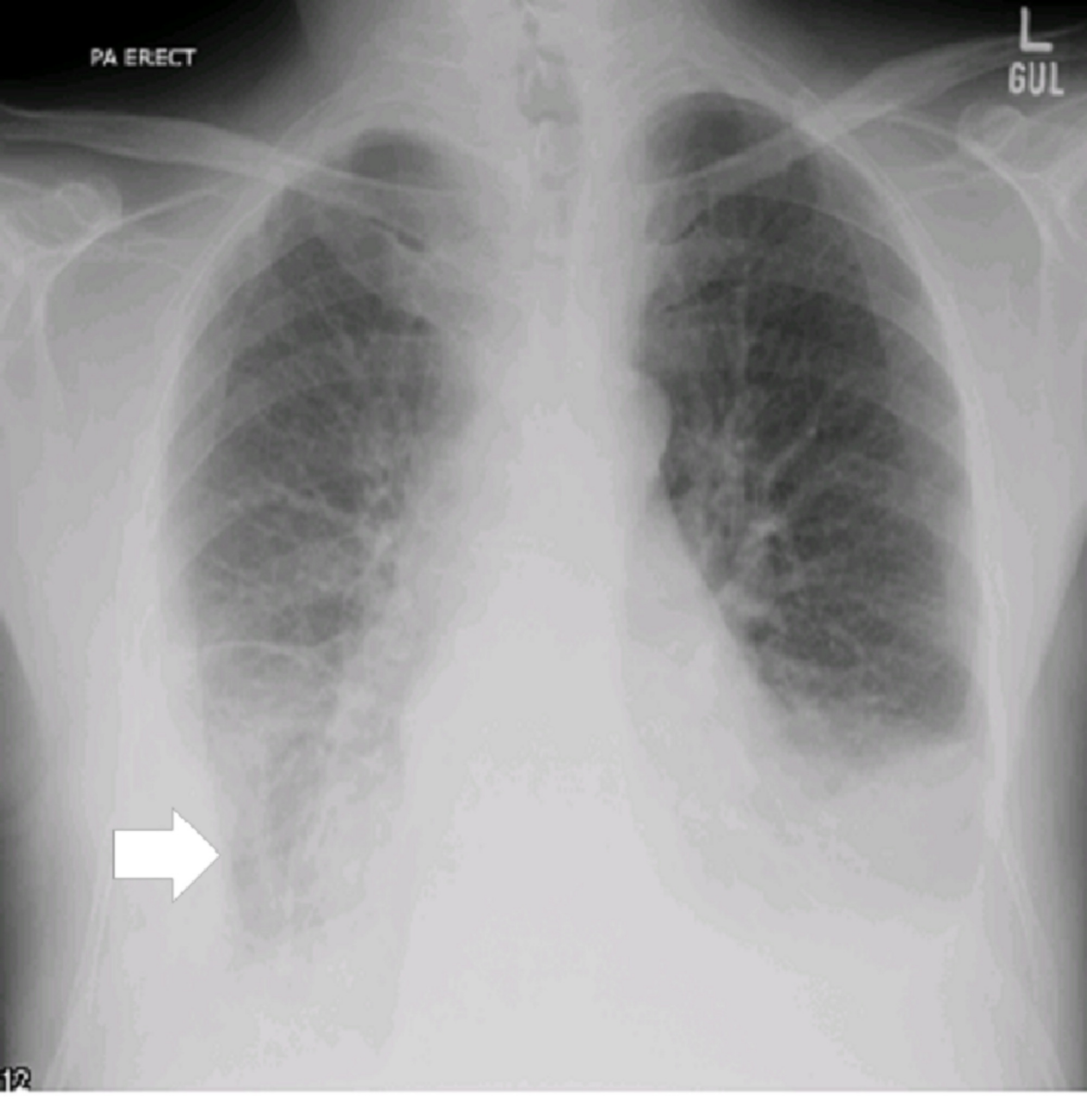
Image showing the chest X-ray Right-sided pleural effusion with the angle obiterated (white arrow)

One month later, the patient presented to the emergency department again with complaints of shortness of breath along with a dry cough. He had a pulse of 90 bpm, respiratory rate 20/minute, BP 121/74, and on auscultation, he had decreased breath sounds on the right side along with pitting edema in the lower extremities. Radiological imaging revealed right-sided loculated effusion (Figure 1).

His pleural fluid analysis revealed a protein content of 3.4 g/dl, glucose of 91 mg/dl, and a white blood cell count of 1300 cells/mm^3^ with 90% lymphocytes. Therapeutic thoracentesis was performed again and 1100 ml of fluid was drained. Post tap imaging revealed fibrous bands on the right side of the chest X-ray. Two-dimensional (2D) echocardiogram revealed that both the atria were mildly dilated and the left ventricular systolic function was preserved with an ejection fraction of 55%. There were prominent septal bounce and significant respiratory variation in the mitral and tricuspid inflow velocities. Pulmonary vein Doppler showed prominent diastolic flow consistent with increased left atrial pressure. The thickened pericardium was also visible on M-mode. These findings, in correlation with the clinical picture, suggested the diagnosis of constrictive pericarditis. The patient was advised a cardiac magnetic resonance imaging (MRI) and pericardial stripping was performed the following day. The patient completed his anti-tuberculous therapy and is now symptom-free. He was scheduled for follow-up after two weeks.

Case 2

A 52-year-old male, known case of diabetes mellitus and hypertension, presented to the pulmonology clinic with complaints of dyspnea, bilateral swelling of the feet, non-productive cough, and undocumented weight loss.

**Figure 2:**
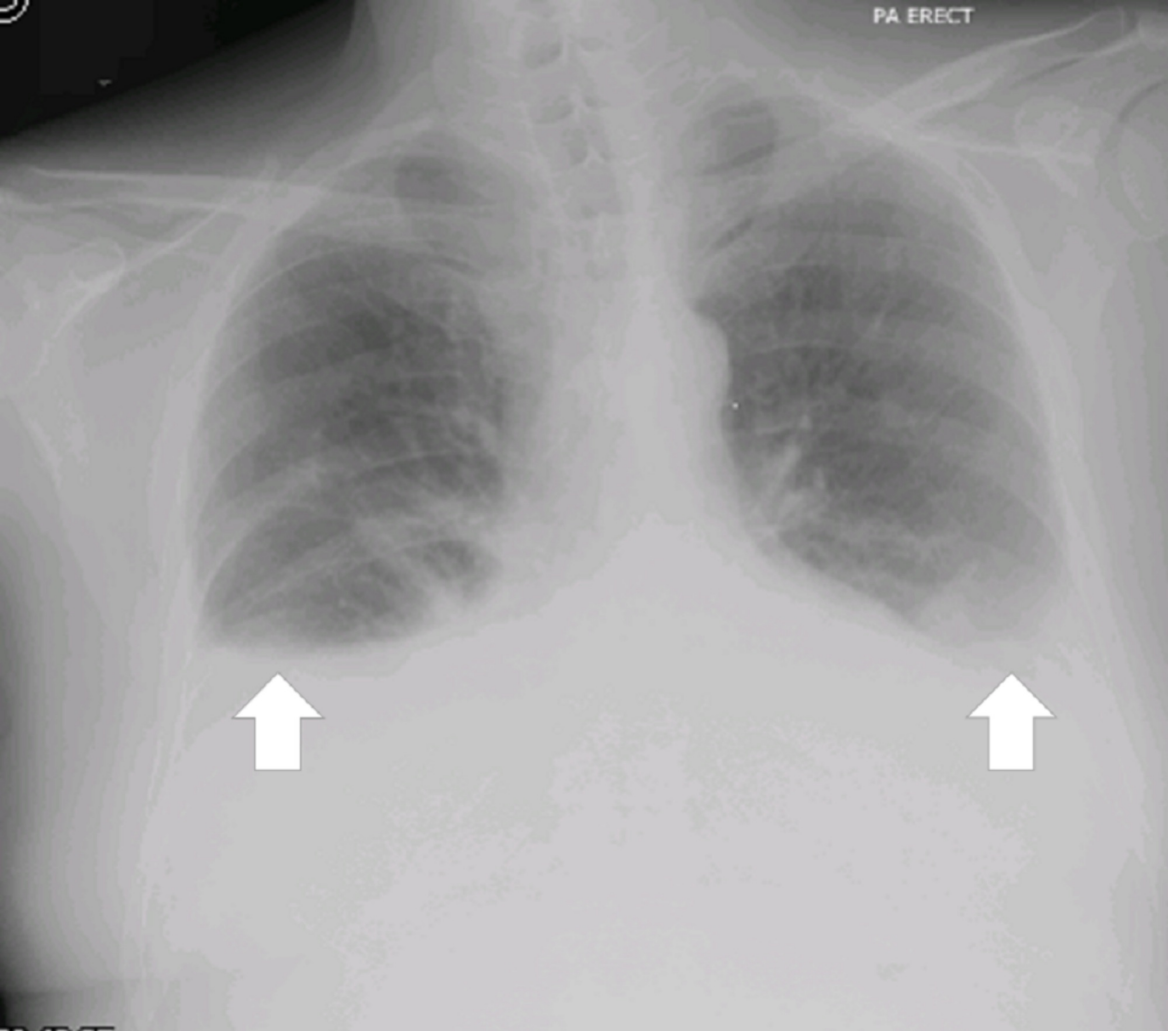
Image showing the chest X-ray Bilateral homogeneous, pulmonary effusions with pulmonary vascular congestion (white arrows)

He had a previous history of TB contact. On examination, the patient was vitally stable with a BP of 124/84, pulse 88 bpm, respiratory rate of 20/minute, and was afebrile. The trachea was central. Bilateral basal crepitations on chest auscultation were heard and were more marked on the right side. Bilateral pedal edema was also present. The rest of the systemic examination was unremarkable. Radiological imaging showed bilateral pleural effusion (Figure 2).

**Figure 3:**
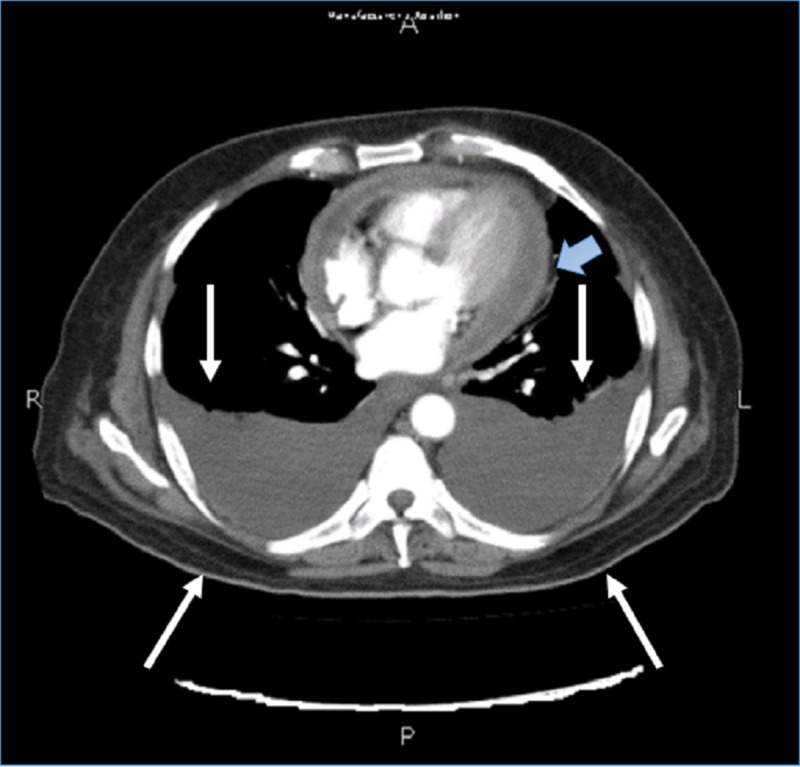
Image showing computerized tomgraphy scan Bilateral opacities that are consistent with pleural effusions (white arrows). Image also shows pericardial thickening, indicated by the blue arrow.

AFB smears were negative. A suspicion of constrictive pericarditis was raised. Computerized tomography (CT) (Figure 3) of the chest showed moderate bilateral pleural effusion, significant pericardial thickening, and enhancement, with minimal pericardial effusion. 

Pressure effects were also visible on the liver with venous congestion and delayed hepatic vein filling. A transthoracic echocardiogram was ordered, which showed normal-sized cardiac chambers, a normal left ventricular systolic function, and grade I left ventricular diastolic dysfunction. The report was suggestive of normal left ventricular (LV) filling pressure. A significant respiratory variation of the E wave was noted at the mitral valve. A dilated inferior vena cava with loss of inspiratory collapse was also noted. Trace to mild circumferential pericardial effusion with significant pericardial thickening was present.

Again, these features suggested constrictive pericarditis. The patient was acutely managed with diuretics and advised further workup to establish the diagnosis. However, the patient was lost to follow-up.

Subsequently, the patient presented to the emergency department after three months, with an exacerbation of shortness of breath. On examination, he was hemodynamically stable but had a respiratory rate of 25/minute. Chest auscultations revealed bilateral basal crepitations. At this time, his chest X-ray showed a right-sided pleural effusion. Pleurocentesis was done. His pleural fluid analysis revealed a protein content of 3.2 g/dl, glucose of 441 mg/dl, LDH of 177 IU/l, and a white blood cell count of 220 cells/mm^3^ with 55% lymphocytes. The AFB smear was negative. Cytology was also negative for any malignancy. Cardiothoracic surgery was taken on board and the patient was advised emergent pericardiectomy. However, the patient preferred to undergo an elective procedure and the cardiology team was involved to optimize his clinical condition with regards to shortness of breath.

Five days later, the patient underwent pericardiectomy. Postoperatively, the patient dropped blood pressures and required high inotropic support. This was followed by a further deterioration of renal function, and he required continuous renal replacement therapy. Due to a suspicion of cardiac tamponade, he underwent sternotomy again. However, no evidence of tamponade was observed. The patient’s condition further deteriorated and despite our best attempts at resuscitation, he did not survive. Pericardial histopathology showed dense chronic inflammation and fibrosis compatible with the clinical diagnosis of constrictive pericarditis.

## Discussion

A large proportion of the pleural effusion presentations are due to congestive heart failure, malignancy, pneumonia, or pulmonary emboli. Bilateral pleural effusions can be caused by liver or renal failure, hypothyroidism, hypoalbuminemia, and constrictive pericarditis [5].

Pleural effusion occurs in about 50% of patients with constrictive pericarditis [6], and several mechanisms have been proposed for its occurrence. The diastolic dysfunction of the left ventricle might cause elevations in the intravascular hydrostatic pressure, leading to a transudative pleural effusion. A similar mechanism may cause systemic venous congestion and ascites. This abdominal collection of fluid can later accumulate in the pleural cavity by tracking through defects in the diaphragm. Exudative pleural effusions are primarily inflammatory in etiology and may result from a variety of infections, post-cardiac-injury syndrome, or radiotherapy.

TB constitutes 6% of the cases of constrictive pericarditis around the world, making it a rare presentation [7]. However, in underdeveloped countries, it is the leading cause of constrictive pericarditis. Other causes include purulent infections, trauma, cardiac surgery, and acute viral and idiopathic pericarditis [2,8]. The first patient presented in this series developed constrictive pericarditis with concurrent active TB. According to the literature [7], and our own experience, it takes time for a patient with active tuberculosis to develop constrictive pericarditis, which is, thus, a late complication of the disease process. It is, therefore, rare to find concurrent active tuberculosis in a patient with constrictive pericarditis. Our case adds to the existing literature on the rare presentations of this common infection. An extensive literature review yielded one similar case of concurrent active tuberculosis in a patient with constrictive pericarditis reported by Liu et al. [9].

Constriction causes a defect in the heart's diastolic filling due to reduced diastolic compliance resulting in decreased cardiac output. Presenting symptoms in constrictive pericarditis are, hence, very similar to congestive heart failure with peripheral edema, dyspnea, weight gain, ascites, and pleural effusions. Raised jugular venous pressure may be a prominent physical sign, which would further increase during inspiration. The other important physical sign of constrictive pericarditis is a pericardial knock, a diastolic sound that occurs 0.05–0.10 seconds after the aortic component of the second heart sound, and usually before the third heart sound.

Unexplained pleural effusion, especially in conjunction with a raised jugular venous pressure, the presence of Kussmaul's sign, pedal edema, and ascites, should alert the physician to the possibility of constrictive pericarditis.

Left-sided pleural effusion is more characteristic of constrictive pericarditis; however, bilateral pleural effusions can also be an initial presentation [10]. Our patients presented with bilateral pleural effusion. Later on, in the disease course, the second case showed a right-sided pleural effusion. This is similar to the findings of Tse G et al. [2], demonstrating a bilateral pleural effusion, but contradicting the unilateral pleural effusion with constrictive pericarditis reported by Ali A et al. [11] as well as Haw A et al. [12]. Our cases, therefore, are unique in presentation and disease course.

## Conclusions

Constrictive pericarditis can be treated conservatively or surgically; however, the definitive treatment of constrictive pericarditis is surgical pericardiectomy. Although a small proportion of pleural effusion is attributable to constrictive pericarditis, management principles depend largely on correct diagnosis.

In conclusion, we suggest putting constrictive pericarditis as the differential when the etiology of pleural effusion is ambiguous, and the patient has undergone multiple pleural taps.
